# Association among B lymphocyte subset and rheumatoid arthritis in a Chinese population

**DOI:** 10.1186/s13018-021-02883-8

**Published:** 2021-12-20

**Authors:** Haiyan You, Mengwei Cheng, Cui Ma, Wenjuan Zheng, Yu Jiang, Di Chen, Yu Tang

**Affiliations:** 1 Department of Laboratory Medicine, Affiliated Hospital of Jiangsu University, Zhenjiang, Jiangsu China; 2 Department of Rheumatology, Affiliated Hospital of Jiangsu University, Zhenjiang, Jiangsu China

**Keywords:** B lymphocyte, Inflammation, Rheumatoid arthritis, Chinese population

## Abstract

**Background and aim:**

Autoantibody production are the main risk factors for inflammation of rheumatoid arthritis (RA). This study aimed to investigate differences in B lymphocyte subsets (native B, memory B, and plasmablasts) and several cytokines in RA patients and their correlation with the clinical parameters.

**Methods:**

In total, 81 RA patients (active RA and inactive RA) and 40 healthy subjects were recruited between September 2018 and October 2020. The distribution of B lymphocyte subsets in peripheral blood samples was measured via flow cytometry and the plasma cytokines were detected by enzyme linked immunosorbent assay. The receiver operating characteristic curve (ROC) was used to evaluate the value of each index for RA diagnosis and activity prediction.

**Results:**

The percentages of native B and memory B cells in RA patients did not differ significantly from the percentages of those in healthy controls. However, the percentage of plasmablasts in active RA patients was significantly higher compared with healthy subjects and inactive RA patients. The percentage of plasmablasts was significantly related to C reaction protein. ROC curve analysis showed that when the best cutoff value of plasmablasts/B cell was 1.08%, the area under the curve (AUC) for diagnosing RA was 0.831 (95% CI 0.748 ~ 0.915), the specificity was 91.4%, and the sensitivity was 67.5%. The AUC predicted by the combination of plasmablast and anti-CCP for active RA patients was 0.760, which was higher than that of plasmablast and anti-CCP.

**Conclusion:**

In conclusion, the percentage of plasmablast varies among RA patients in different stages. The percentage of plasmablasts can be used as an early diagnosis marker for RA.

## Introduction

Rheumatoid arthritis (RA) is a common inflammatory arthritis [1], with a prevalence of 0.5–1.0% [2]. RA in the active stage causes cartilage and bone damage, disability, and other comorbidities [2], which lead to a heavy burden on individual and society [3]. Many studies have made great efforts to establish early diagnosis and optimize treatment plans to control inflammation and reduce or prevent the progression of RA patients. Environmental factors, genetic factors, as well as gene–environment interactions have been proved to be associated with RA risk [4]. Data from a study conducted in twins suggested that genetic factors account for approximately 60% of the risk of RA [5].

It is well known that inflammation constitutes a major risk factor for bone destruction. A large number of cytokines, such as tumor necrosis factor alpha (TNF-α) and interleukins (IL)-1, 6, and 15, were found in the joint fluid and blood of patients with active diseases [6]. However, few studies throw light on the association between the levels of IL-18 and RA. Additionally, the inflammatory infiltrate in RA includes T lymphocytes, B lymphocytes, monocytes, and dendritic cells [7, 8]. RA patients develop periarticular osteopenia and joint erosions early in the disease process [9]. B cells in RA can exert pathogenic effects through autoantibody production and cytokine production [10]. B cells are activated by activated T helper cells to become plasma cells, which can secrete large amounts of immunoglobulins, including rheumatoid factor (RF) and other antibodies, further causing inflammation and destruction of joints. Human B lymphocytes in peripheral blood can be divided into three subgroups representing different stages of differentiation: native B cells, memory B cells, and plasmablasts. Several studies have found the inconsistent results with regard to B cell subset, and the transitional B and native B cells increased in systemic lupus erythematosus compared with healthy controls [11, 12]. There were few studies underlying their association between B cell subsets and RA.

Therefore, our group investigated the differentiation status of B lymphocytes in the peripheral blood of RA patients, compared its relationship with the clinical characteristics and laboratory-related indicators, and further evaluated its role in the mechanism of RA.

## Patients and methods

### Study subjects

In all, 81 RA patients were consecutively recruited from the Affiliated Hospital of Jiangsu university between September 2018 and October 2020. RA patients were diagnosed according to the criteria of the American College of Rheumatology (1987) [13]. The 40 healthy controls were ethnicity, age, and sex matched, free from any family history of RA through health check-ups. The individuals with cancer, leukemia, and other rheumatic diseases were excluded from the study. All participants were interviewed, and records on age, sex, onset age, disease duration, treatment duration, rheumatoid factor (RF), anti-cyclic citrullinated peptide (anti-CCP), C-reactive protein (CRP), RA disease activity score (DAS28), and functional class. All procedures were in compliance with the Helsinki declaration, and informed consent was obtained from all patients and participants before inclusion in the study. Ethical approval was obtained from the Ethics Committee of Affiliated Hospital of Jiangsu University (ID: SWYXLL2200121-5).

### Determination of B lymphocyte subsets

The participants provided 2 mL of peripheral blood in ethylene diamine tetraacetic acid (EDTA) tubes. The blood sample was mixed by turning the vials upside down 3–5 times, and 100 μL of peripheral blood was drawn into a flow tube. Further, 5 μL CD38-FITC, 5 μL CD27-PE, and 1.25 μL CD19-APC (BD, San Jose, California, USA) was added to the flow tube, followed by shaking, mixing, and then incubating for 15 min at 20–25 ℃; then, 450 μL 1X hemolysin was added, and the mixture was incubated for 15 min at 20–25℃ after shaking and mixing. Following this, 2 mL PBS, was added, followed by centrifugation at 1500 rpm for 5 min. The supernatant was discarded, and the procedure was repeated twice. Subsequently, 200 μL PBS was added to resuspend the pellet, and the sample was ready to be tested. In all, 5000 events within CD19^+^ gate were acquired via FACSCalibur flow cytometry and analyzed by with FlowJo software (BD Biosciences, CA, USA). B lymphocyte subgroups were grouped and gated according to their characteristic forward-scatter and side-scatter and their expression status for CD19, CD38, or CD27: native B (CD19 + CD27-CD38-/ +), memory B (CD19^+^CD27^+^CD38^−^), and plasmablast (CD19^+^ CD27^++^CD38^++^).

### Determination of IL-6 and IL-18 expression level

The Human IL-6 and IL-18 ELISA kits were purchased from Beyotime Co. Ltd (Shanghai, China). The plasma IL-6 and IL-18 levels of RA patients and controls were measured according to the standard experimental procedure. We draw the standard curve with the concentration of the standard substance as the abscissa and the absorbance at 450 nm as the ordinate. The plasma levels of IL-6 and IL-18 were calculated by referring to a standard curve.

### Statistical analysis

The Student’s test and One-Way ANOVA were used for Continuous variables (e.g. means ± standard deviation) comparison between two or more groups. Similarly, qualitative data (e.g. frequencies and percentages) were evaluated by chi-squared test. Pearson’s correlation was adapted to correlate the studied parameters. The receiver operating characteristic curve (ROC) and the area under the curve (AUC) were used to analyze the diagnostic efficacy of related indicators for RA. All statistical analyses in this study were conducted using the statistical package for social sciences (SPSS) software package (var. 22.0; IBM, Armonk, New York, USA). *P* < 0.05 was considered to indicate statistically significant.

## Results

### Characteristics of the study population

Table 1 provides the baseline characteristics of participants. The mean age and the distribution of sex of RA patients and healthy subjects did not differ significantly (P = 0.363 and 0.549, respectively). There were significant differences between RA patients and controls with regard to IL-6 and IL-18 levels. The positive rates of RF and CCP antibodies in RA patients were 80.2% and 56.8%, respectively. The functional classes for I, II, III, and IV were 7 (8.6%), 35 (43.2%), 27 (33.3%), and 12 (14.8%), respectively.

**Table 1:** Patient demographics and risk factors in rheumatoid arthritis

Variable	Cases (n = 81)	Controls (n = 40)	*P*
Age (years)	56.70 ± 14.02	54.35 ± 11.84	0.363
Female, no. (%)	57 (70.4%)	26 (65.0%)	0.549
IL-6 (pg/mL)	71.28 ± 29.81	20.59 ± 9.65	** < 0.001**
IL-18 (pg/mL)	131.34 ± 48.24	49.55 ± 9.22	** < 0.001**
Age at onset, years, mean ± SD	44.06 ± 10.04		
RF-positive, no. (%)	65 (80.2%)		
Anti-CCP positive, no. (%)	46 (56.8%)		
CRP, mg/L	33.01 ± 40.61		
DAS28	4.31 ± 1.94		
Functional class, no. (%)			
I	7 (8.6%)		
II	35 (43.2%)		
III	27 (33.3%)		
IV	12 (14.8%)		

Bold values are statistically significant (*P* < 0.05)

RF, Rheumatoid factor; Anti-CCP, anti-cyclic citrullinated peptide; CRP, C-reactive protein; ESR, Erythrocyte sedimentation rate; DAS28, rheumatoid arthritis disease activity score

### B lymphocyte subsets, and RA disease

**Fig. 1:**
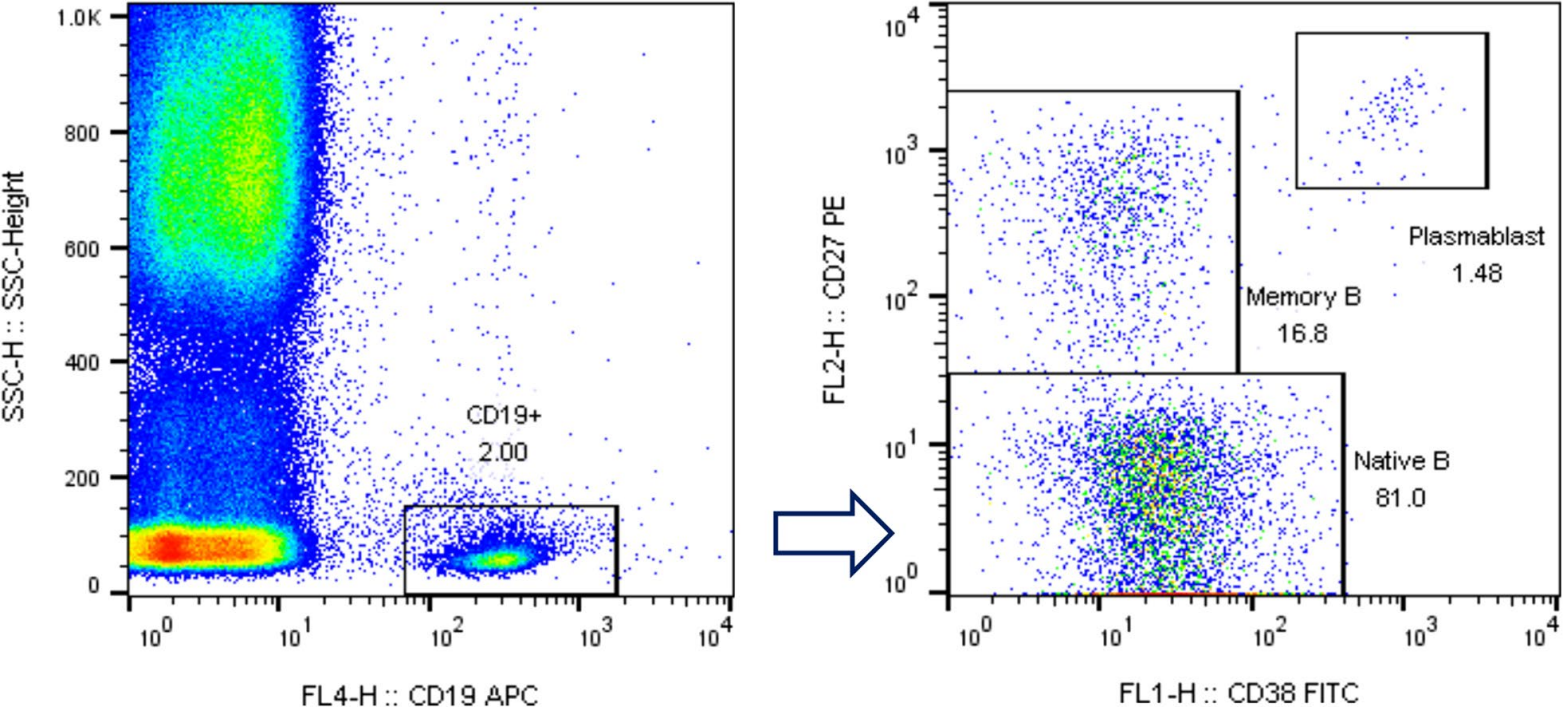
Flow cytometry gating strategy for measuring CD27 and CD38 expression in CD19^+^B cells

The study investigated the distribution of B lymphocyte subsets (native B, Memory B, and plasmablasts) on CD19 + B cells. The gating strategy of flow cytometry is shown in Fig. 1. Our results indicated that there were no differences between RA inactive, RA active and control individuals with regard to the percentage of native B and memory B cells (*P* < 0.05, Table 2). Notably, the proportion of plasma cells in patients with active RA was significantly higher than that in patients with inactive RA and the normal individuals (active RA vs. inactive RA vs. control: 2.86 ± 3.39 vs. 1.45 ± 0.42 vs. 0.95 ± 0.51, Table 2). Moreover, there was significant difference in the proportion of plasma cells between the inactive RA group and the normal group (*P* < 0.05, Table 2). Subgroup analysis was conducted according to age, RF levels, anti-CCP levels, and function class (Table 3). Furthermore, no significant differences were found between RF-positive group and RF-negative group with regard to B lymphocyte subsets. This result was also observed in the anti-CCP and function class subgroup.

**Table 2:** B lymphocyte subsets in participants

Variable	Inactive RA	ActiveRA	Control	*P* value
	N = 25	N = 56	N = 40	
Native B/CD19 + B(%)	65.32 ± 17.46	63.45 ± 15.99	62.69 ± 14.35	0.805
Memory B/CD19 + B(%)	33.02 ± 17.47	33.40 ± 15.24	35.28 ± 14.16	0.795
Plasmablast/CD19 + B(%)	1.45 ± 0.42*	2.86 ± 3.39*^,#^	0.95 ± 0.51	** < 0.001**

Native B cell markers: CD19^+^CD27^−^CD38^−/+^; Memory B cell markers: CD19^+^CD27^+^CD38^−^; Plasmablast cell markers: CD19^+^CD27^++^CD38^++^

*vs. control; ^#^vs. inactive RA

Bold values are statistically significant (*P* < 0.05)

**Table 3:** Subgroup analysis of B lymphocyte subsets in rheumatoid arthritis

Variable	Native B/CD19 + B (%)	Memory B/CD19 + B (%)	Plasmablast/CD19 + B (%)
Age			
Male (n = 24)	65.34 ± 16.68	31.55 ± 16.16	2.79 ± 3.33
Female (n = 57)	63.47 ± 16.36	34.01 ± 15.80	2.27 ± 2.70
* P* value	0.643	0.528	0.458
RF			
Negative (n = 16)	64.04 ± 14.13	33.48 ± 13.73	2.23 ± 2.68
Positive (n = 65)	64.02 ± 16.98	33.23 ± 16.42	2.47 ± 2.96
* P* value	0.771	0.956	0.996
Anti-CCP			
Negative (n = 35)	62.19 ± 18.55	35.41 ± 18.00	2.14 ± 2.67
Positive (n = 46)	65.42 ± 14.56	31.66 ± 13.98	2.63 ± 3.06
* P* value	0.452	0.294	0.381
Function class			
I + II (n = 42)	64.83 ± 15.47	32.56 ± 14.93	2.36 ± 2.89
III + IV (n = 39)	63.16 ± 17.46	34.05 ± 16.94	2.49 ± 2.92
* P* value	0.839	0.676	0.650

Native B cell markers: CD19^+^CD27^−^CD38^−/+^; Memory B cell markers: CD19^+^CD27^+^CD38^−^; Plasmablast cell markers: CD19^+^CD27^++^CD38^++^; RF, Rheumatoid factor; Anti-CCP, anti-cyclic citrullinated peptide

**Fig. 2:**
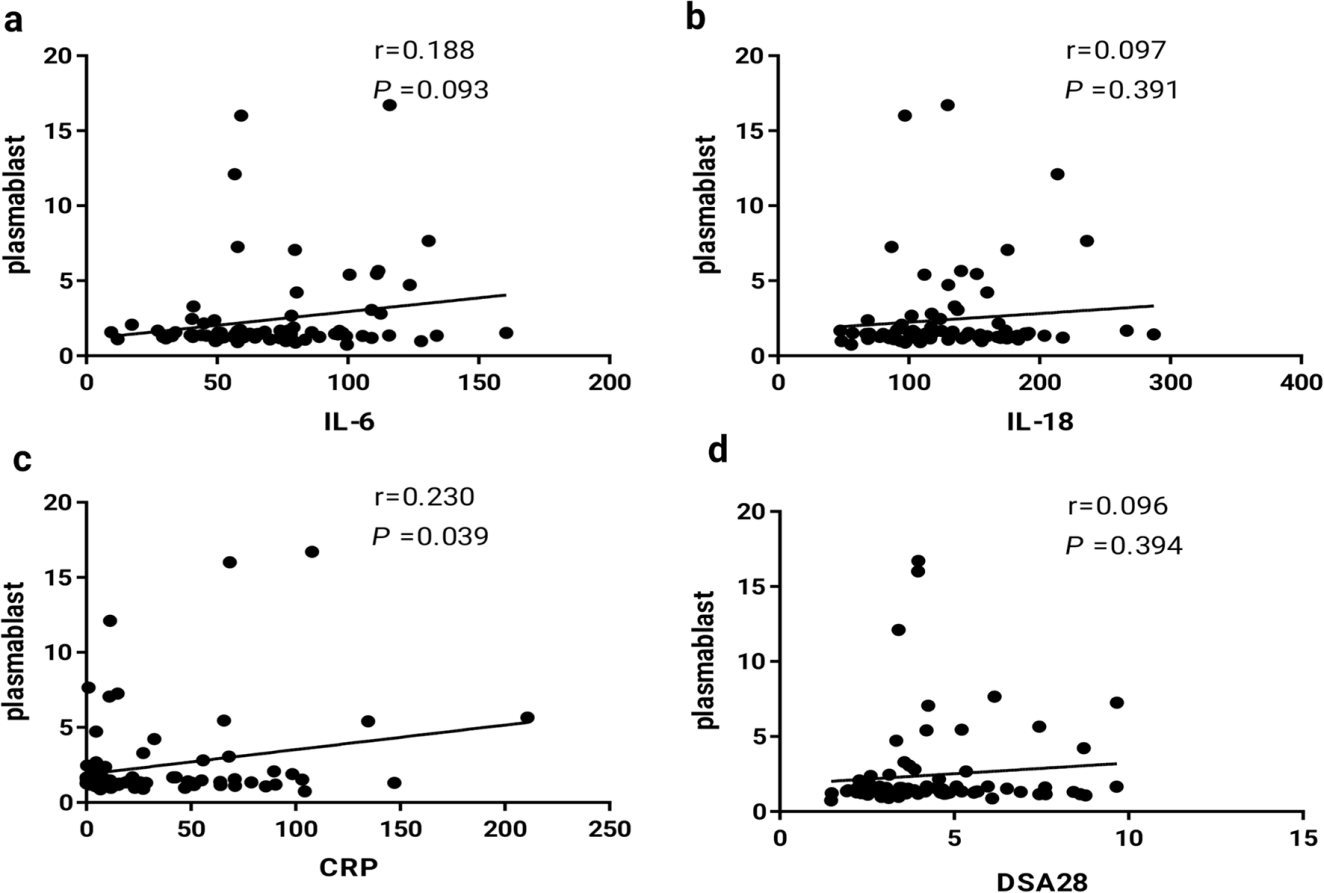
Correlation between the proportions of plasmablasts in the peripheral blood of RA patients and other clinical parameters. **a** IL-6 and plasmablasts, **b** IL-18 and plasmablasts, **c** CRP and plasmablasts, and **d** DAS28 and plasmablasts

To further verify the association between plasmablasts and clinical indicators, we analyzed the correlation between IL-6, IL-18, CRP, and DAS28 scores, and found that the proportion of plasmablasts showed no correlation with IL-6, IL-18, and DAS28, but had a significant correlation with CRP (Fig. 2). This indicated that plasmablasts are not closely associated with disease activity and can’t be used as prognostic indicators for RA.

### The diagnostic and activity prediction performance of the percentage of plasmablasts in RA

**Fig. 3:**
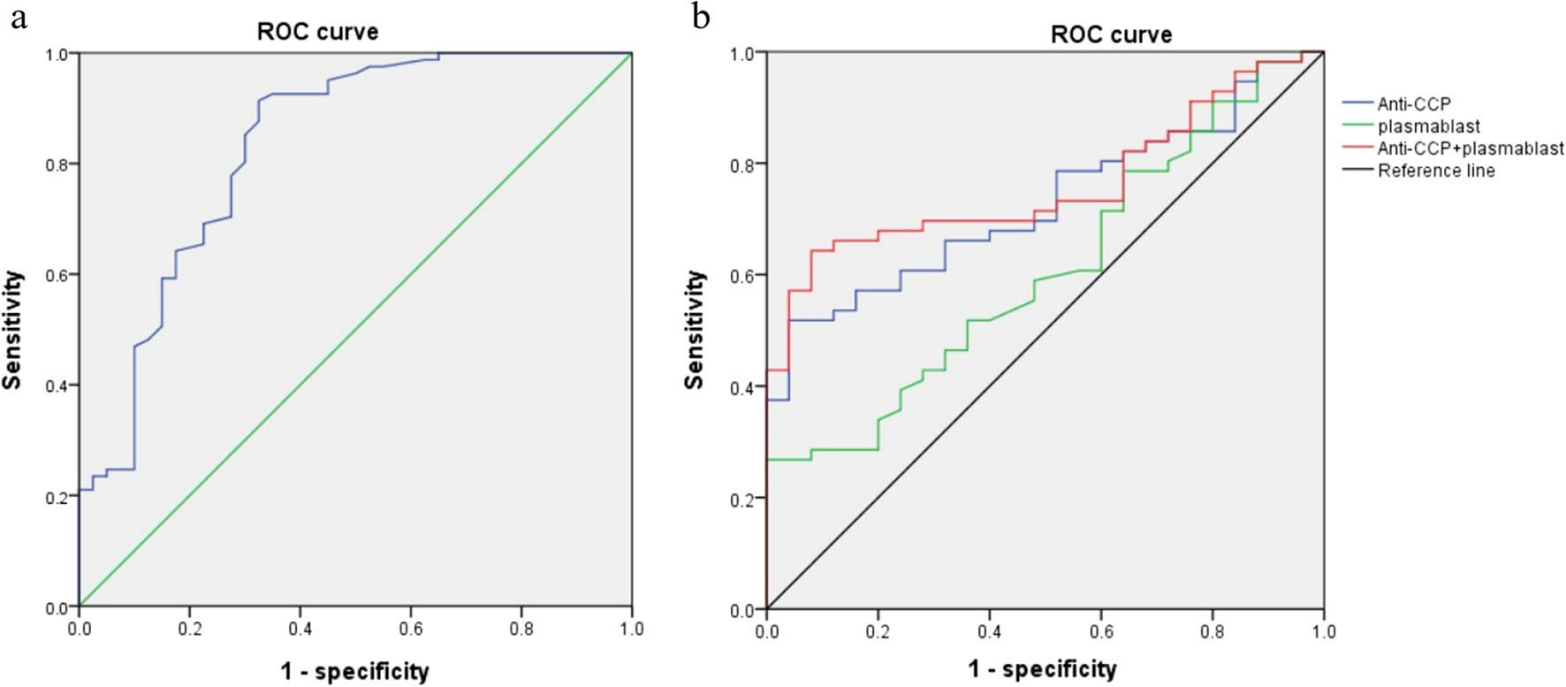
Receiver operating characteristic curve of various indicators for diagnosis and activity prediction of RA. **a** The proportion of plasmablasts for RA diagnosis, **b** The proportion of plasmablast and anti-CCP for RA activity

When the optimal critical value of the percentage of plasmablasts was 1.08%, the AUC for the diagnosis of RA was the highest (0.831), the specificity was 67.5%, and the sensitivity was 91.4% (Table 4, Fig. 3). The AUC predicted by plasmablast and anti-CCP for active RA patients were 0.607 and 0.728, respectively, and the combined predicted AUC was 0.760, which was higher than that of plasmablast and anti-CCP (Table 4, Fig. 3). Thus, the level of plasmablast and anti-CCP could be viewed as potential biomarkers for the early diagnosis of RA and its disease activity.

**Table 4:** Diagnostic efficacy of various indicators for the diagnosis of RA and its disease activity

Indicator	AUC	95%CI	Optimal threshold	Sensitivity(%)	Specificity(%)	Youden index
RA vs. control						
Plamablast/CD19 + B, %	0.831	0.748–0.915	1.08	91.4	67.5	0.589
Active RA vs. inactive RA						
Plamablast/CD19 + B, %	0.607	0.479–0.734	2.57	26.8	100.0	0.268
Anti-CCP, U/mL	0.728	0.621–0.835	21.66	51.8	96.0	0.478
Combination	0.760	0.658–0.862	Plasmablast > 1.51%	64.3	92.0	0.563
			Anti-CCP > 25.88 U/mL

## Discussion

T cells, B cells (antigen presentation and autoantibody production) and the orchestrated interaction of pro-inflammatory cytokines (TNF-α, IL-6, and IL-1) play key roles in the pathophysiology of RA [14, 15]. B cell depletion was demonstrated to be a successful treatment in RA [16]. The rituximab therapy (a monoclonal antibody against CD20-positive B cells) was used to depleted B cells in RA patients. Incomplete depletion of B cells is associated with a poor prognosis [17]. Therefore, it is necessary to detect B cell subpopulations in RA patients to determine the status of B cell exhaustion. Furthermore, IL-18 contributes to inflammation and angiogenesis in RA by inducing leukocyte extravasation and releasing chemokines from RA synovial fibroblasts [18]. Therefore, in this study, we focus on the B lymphocyte subsets and several important cytokines (IL-6 and IL-18).

At present, there is no uniform standard for the definition of B lymphocyte subsets in domestic and foreign research. Several studies have divided B cell subgroups into four categories, namely native B cells (IgD^+^ and CD27^−^); pre-switch cells (IgD^+^ and CD27^+^); memory B cells (IgD^−^ and CD27^+^); and plasmablasts (IgD^−^ and CD27^++^) [19–21]. Rehnberg et al. investigated the percentage of B cell population: CD38^++^CD24^++^IgD^±^ as immature/transitional B cells, CD38^+^IgD^+^IgM^++^CD24^+^CD27^−^ as native B cells, CD38^+^IgD^−^CD24^−^CD27^+^ as mature B cells, and CD38^+++^IgD^−^CD27^+^ as plasmablasts [22]. The study chose surface markers (CD19, CD27, and CD38) based on previous studies to label B lymphocytes, and divided B cells into native B cells (CD19^+^CD27^−^CD38^−/+^), memory B cells (CD19^+^CD27^+^CD38^−^), and plasmablasts (CD19^+^CD27^++^CD38^++^) to explore the distribution of B cell subsets in RA patients at different disease stages.

Potter et al. found that there was no significant difference in either the percentage or number of naive B (CD19^+^CD27^−^), memory B (CD19^+^CD27^+^), and early plasma cells (CD19 + CD27^high^) between RA patients and healthy controls [23]. The results of Fedele et al. revealed that very early-RA and early-RA patients showed higher percentages and numbers of naïve B cells (IgD^+^CD27^−^) compared with long-standing RA patients among a population of 123 Italian RA patients [24]. However, the opposite results were found for double negative (IgD^−^CD27^−^) memory B cells and plasmablasts (CD38^+^CD27^+^). This study indicated that most B lymphocyte subsets were not significantly different in the peripheral blood between RA patients and normal controls, but the percentage of plasmablasts in the RA active group was significantly higher than that in the normal control group and the RA inactive group (P < 0.05). It suggests that the percentage of plasmablasts may be related to disease activity. The reasons for the divergence are as follows: first, the study subjects were of different races; second, the selection of surface antibodies for B cell differentiation were inconsistent; and third, the manufacturer and clone number of the flow reagent are different.

The proportion of plasma cells in RA patients is significantly higher than that in healthy controls. This may be attributed to the fact that plasmablasts produce a large number of different types of autoantibodies, causing tissue damage, and playing a major role in the pathogenesis of RA. In addition, the proportion of plasma cells in active RA was significantly higher than that in inactive RA (*P* < 0.05). However, the proportion of plasma cells is not a suitable marker for predicting the active stage of RA. Clinical heterogeneity, different ethnic populations, and small sample sizes may contribute to these disparities.

Although positive findings were observed, several limitations should be taken into consideration. First, this study is a single-center retrospective study, which cannot simply be applied to other populations. Second, the number of participants was small, which lead to false-negative or false-positive results to a certain degree. Third, confounding factors such as drinking and education level were not considered in the analysis of the results because of lack of original data.

In conclusion, the percentage of plasmablasts in the active RA group is significantly higher than that in the inactive RA group and normal control group and is related to the patient’s laboratory indicators. The percentage of plasmablasts can be used as a marker for early diagnosis of RA. Large-sample studies are also needed to verify its diagnostic efficacy in predicting RA activity.

## Data Availability

The datasets used and/or analyzed during the current study are available from the corresponding author on reasonable request.

## Abbreviations

RA: Rheumatoid arthritis
ELISA: Enzyme-linked immunosorbent assay
ROC: Receiver operating characteristic curve
AUC: Area under the curve
IL-1: Interleukin-1
TNF-α: Tumor necrosis factor alpha
RF: Rheumatoid factor
Anti-CCP: Anti-cyclic citrullinated peptide
CRP: C-reactive protein
DAS28: RA disease activity score
EDTA: Ethylene diamine tetraacetic acid
